# Effects of chloramphenicol, povidone-iodine 1% and 5% eye drops on the colonisation of conjunctival flora in patients undergoing cataract surgery

**DOI:** 10.4314/gmj.v57i2.1

**Published:** 2023-06

**Authors:** Nasrin Tofighi, Mohsen Gohari, Maryam Sadeh, Hosein Fallahzadeh, Fatemeh Jabinian

**Affiliations:** 1 Department of Operating Room and Anesthesiology, School of Paramedicine, Shahid Sadoughi University of Medical Sciences, Yazd, Iran; 2 Department of Ophthalmology, Geriatric Ophthalmology Research Center, Shahid Sadoughi University of Medical Sciences, Yazd, Iran; 3 Department of Laboratory Sciences, School of Paramedicine, Shahid Sadoughi University of Medical Sciences, Yazd, Iran; 4 Department of Biostatistics and Epidemiology, Shahid Sadoughi University of Medical Sciences, Yazd, Iran

**Keywords:** Cataract surgery, Chloramphenicol, Conjunctiva, Povidone-Iodine, Microflora

## Abstract

**Objectives:**

the aim was to compare 2 drops of either 5% chloramphenicol, 1% povidone-iodine or 5% povidone-iodine before cataract surgery on reducing the colonisation of bacterial flora in the conjunctiva.

**Design:**

This was a double-blind, randomised clinical trial study.

**Setting:**

Patients referred to Shahid Sadoughi Hospital in Yazd, Iran, for cataract surgery were studied.

**Participants:**

Totally 260 patients were enrolled.

**Intervention:**

The affected lower fornix was gently sampled with a sterile swab and cultured on appropriate microbiological media. Then one of the 3 solutions mentioned above was instilled into the conjunctival sac of the cases in groups 1, 2 and 3, respectively. After thirty minutes, new conjunctival swabs were taken and cultured.

**Main outcome measures:**

The type of bacteria isolated and their colony-forming unit per mL (CFU/mL) number were primary end-points. The statistical tests of Phi and Cramer's V and Wilcoxon and Kruskal-Wallis were applied to evaluate the relationship between the studied variables and culture results as the secondary end-point.

**Results:**

The studied patients were 129 (49.6%) males and 131 (50.4%) females. Bacterial growth was observed in 49 cases (18.85%); the most commonly isolated bacteria were *Staphylococcus epidermidis* (71.42%). In the povidone-iodine 5% and chloramphenicol groups (but not the povidone-iodine 1%), the decrease in the number of CFU/mL was statistically significant (P = 0.032 and P = 0.005, respectively, Wilcoxon test).

**Conclusion:**

A single dose of povidone-iodine 5% and chloramphenicol effectively reduces the colonisation of normal conjunctival bacteria and can be used as effective prophylaxis.

**Funding:**

This study was part of an MSc thesis of Nasrin Tofighi. Shahid Sadoughi University of Medical Sciences, Yazd, Iran, funded this work.

## Introduction

One serious complication of cataract surgery is endophthalmitis.^1^ Its incidence projects the prevalence of the complication following cataract surgery to be between 0.03 to 1.02.^2^ Today, on account of advances in surgical techniques and preoperative prophylaxis, there is a significant reduction in the incidence of postoperative endophthalmitis.

Much as this complication is rare, it is yet one of the most dangerous intraocular surgery complications due to its high rate of blindness and adverse effects on quality of life.^3^ Studies have shown that the primary source of endophthalmitis following eye surgery is the microflora in the patient's conjunctiva and eyelids.^4^, ^5^ During cataract surgery, a path opens up to the eye's anterior chamber through which the conjunctival lavage fluid containing microflora may enter.^6^

There are several guidelines for managing eye infections during cataract surgery. One of the strategies to lower endophthalmitis risk is to reduce the bacterial flora of the conjunctiva and eyelids.^6^

Some studies have found that topical antibiotics for one to three days before surgery or using povidone-iodine effectively reduce the bacterial flora in the conjunctiva.^4^ Different concentrations of povidone-iodine, that is, between 0.01% to 10% and standard povidone-iodine 5%, are used for this purpose^7^; however, the concentration of povidone-iodine which has the best effect on reducing the bacterial flora of the conjunctiva and eyelid without inducing toxicity is unclear.^4^,^7^,^8^ Some studies have identified that high concentrations impact the corneal epithelium in rabbits' eyes. In addition, 2.5% and 5% of povidone-iodine induce oedema in the rabbit cornea, but this does not occur at 1% or 0.5% concentrations. Therefore, an attempt is made to use povidone-iodine in lower concentrations.^9^ Despite numerous studies on preventing endophthalmitis, the best prophylactic method that can contain postoperative endophthalmitis is not precisely known; further studies are thus needed to determine a rapid and effective procedure for preoperative prophylaxis.^9^

Based on this, we decided to compare the effects of prophylactic chloramphenicol and povidone-iodine 1% and 5% in reducing the colonisation of the conjunctival flora in patients undergoing cataract surgery.

## Methods

This double-blinded, randomised clinical trial study was conducted after approving the plan and obtaining permission from the Research Council of Shahid Sadoughi University of Medical Sciences with IR. SSU. SPH. REC.1398.074 and receiving clinical trial code No.IRCT20190924044870N1 registered on the site: www.irct.ir.

After obtaining written informed consent, two hundred and sixty candidates for cataract surgery, who had been referred to Shahid Sadoughi Hospital in Yazd in 2019-2020, were included in the study.

### Inclusion criteria

1. Age over 40 years
2. No history of systemic and ocular infectious diseases
3. Absence of autoimmune diseases and use of immunosuppressive drugs
4. No use of systemic and topical antibiotics in the last 30 days
5. Lack of allergy and sensitivity to antibiotics and povidone-iodine
6. No history of surgery or trauma of the eye
7. No evidence of inflammatory or infectious diseases in the eye
8. Absence of diseases such as conjunctivitis and blepharitis
9. No signs of flu
10. No chronic dacryocystitis, no eyelid abnormalities except ptosis,
11. Absence of corneal ulcer and resultant scars

**Randomisation:** First, the patients admitted to the ophthalmology ward, who had met the inclusion criteria, were included in the study. Informed consent was obtained from all participating adult subjects, and the patients were then randomly (using a random number table) assigned into three intervention groups. There was no negative control group since every patient would have to receive an antibacterial agent before the surgery.

**Figure 1:**
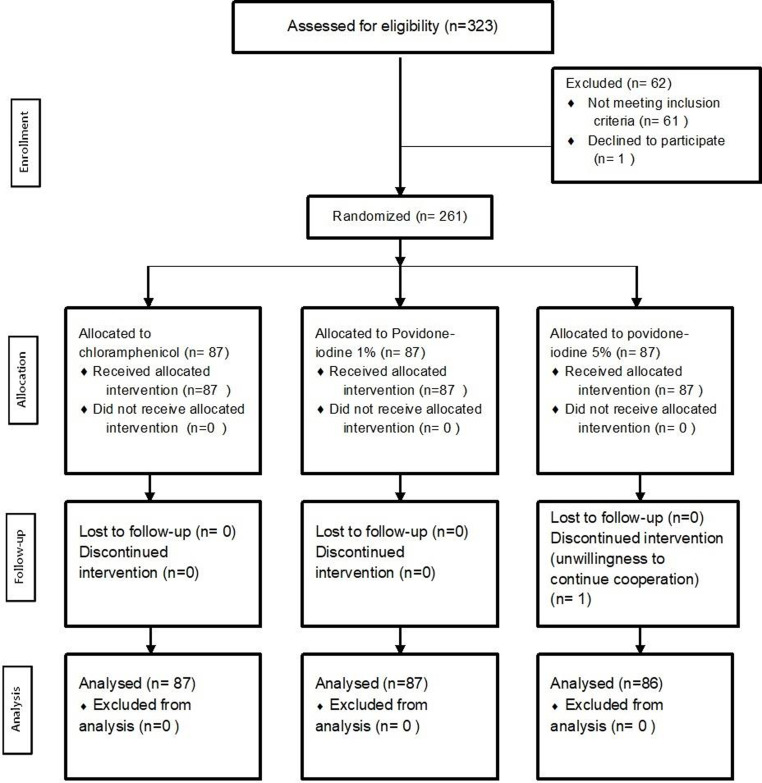
Consort flowchart

**Intervention and Laboratory methods:** First, demographic data, including age, sex, presence/absence of diabetes, and blood glucose levels, were collected from all patients, and then the index eye was sampled by a trained individual before the intervention. For this, the affected lower fornix was gently sampled five times with the sterile swab without local anaesthesia, contact with the other eyelid or touching surrounding skin. About Five minutes following the preparation of the first sample, group 1 received povidone-iodine 5% drops (five cc of distilled water and five ccs of povidone-iodine 10%), group 2 took povidone-iodine 1% drops (nine cc of distilled water and one cc povidone-iodine 10%), and group 3 received chloramphenicol drops (0.5% colo biotic-Sina Daru Tehran-Iran) in their conjunctival sac (Figure 1). All medications used for surgical patients, including eye drops, are commercial, obtained from drug stores and approved for use in the clinical setting by the Ministry of Health in Iran. We did not perform any extra quality checks on them. Thirty minutes after using the previously mentioned drugs, conjunctival specimens were prepared again using the above method and placed on blood agar, chocolate agar, Eosin methylene blue agar (EMB) and thioglycollate broth (all culture media from Merck, Germany) for bacterial culture and incubated at 37°C for 48 hours. The thioglycollate medium (aimed at isolating potential anaerobic bacteria) was incubated for one week. Microbial genera and species were identified by deploying standard microbiological tests used in the microbiology laboratory. The patient was blinded to the type of intervention applied. The operating room nurse who obtained the conjunctival sampling was also blinded to the kind of ophthalmic antibacterial agent used for each patient. The patient was unaware of the type of intervention applied as well. The operating room nurse who obtained the conjunctival sampling was unaware of the kind of ophthalmic antibacterial agent used for each patient. Likewise, the laboratory expert and statistical consultant were blinded to the type of intervention performed.

### Statistical Methods

The collected data were entered into SPSS software version 16 (IBM corporation, Chicago, IL, USA) and analysed, and the relevant statistical tests were performed after checking the normality of the data. Phi and Cramer's V tests were used to evaluate the relationship between the studied variables and culture results. Wilcoxon test was also deployed to compare within-the-group comparisons regarding the change in the number of colony-forming units grown in culture media (CFU). Furthermore, the Kruskal-Wallis test was finally applied to compare the studied groups regarding the number of colonies grown in culture media.

## Results

**Figure 2:**
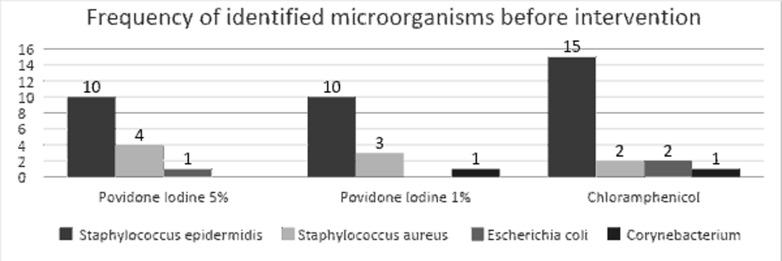
Comparison of isolated bacteria among the three groups

This study was a double-blind, randomised clinical trial involving 260 patients with a mean age of 65.36 ± 10.71 (40 to 91 years). Of the participants, 131 (50.5%) were female and 129 (49.5%) male. The right eyes were studied in 128 patients (49.2%), and the left was studied for the rest. Also, 78 (30%) of the participants presented a history of diabetes. The results of the culture and isolated bacteria from the conjunctiva of the eyes are set out in Figure 2. As evidenced by the diagram, among the microorganisms identified before the intervention, *Staphylococcus epidermidis* (71.42%), followed by *Staphylococcus aureus* (18.36%), had the highest frequency among the positive cultures. In individuals with a positive culture, only one microbial species was isolated; no anaerobic bacteria were isolated.

To evaluate the relationship between age, sex, and history of diabetes with the number of positive cultures, Phi and Cramer's V tests were used and considering p>0.05, no relationship was observed between any of the variables with the number of positive cultures (Table 1).

**Table 1:** The relationship between the studied variables and the number of positive conjunctiva cultures

Variable name N=260	Variable label	Number of positive conjunctiva cultures (percentage)	Number of negative conjunctival cultures (percentage)	P-Value*
**Gender**	Male	23(17.8)	106(82.2)	**0.677**
	Female	26(19.8)	105(80.2)	
**Diabetes mellitus**	Positive	12(15.4)	66(84.6)	**0.350**
	Negative	37(20.3)	145(79.7)	
**Age**	<60	18(20.7)	69(79.3)	**0.447**
	60-70	15(15)	85(85)	
	>70	16(21.9)	57(78.1)	

* P<0.05, Phi and Cramer's V test

Table 2 illustrates the CFU results in each culture for all three groups of 5% povidone-iodine, 1% povidone-iodine and chloramphenicol before and after the intervention using the Kruskal-Wallis test. At a 95% confidence level, no statistically significant difference was identified between the numbers of conjunctiva colonies in the two-culture media in all groups before and after the intervention. Wilcoxon test was used to compare within-the-group comparisons regarding the change in the number of CFU grown in culture media.

**Table 2:** Comparison of changes in mean colony forming units (CFU) between the three groups before and after the intervention

Variable	colony forming units/mL					
	Before intervention			After intervention		
	Povidone-Iodine 5%	Povidone-Iodine 1%	Chloramphenicol	Povidone Iodine 5%	Povidone Iodine 1%	Chloramphenicol
**Blood Agar**						
**n**	86	87	87	86	87	87
**0**	73(84.9)	76(87.4)	69(79.3)	80(93)	80(92)	78(89.7)
**1-10**	8(9.3)	10(11.5)	12(13.8)	6(7)	5(5.7)	7(8)
**11-100**	4(4.7)	1(1.1)	3(3.4)	0(0)	2(2.3)	1(1.1)
**>100**	1(1.2)	0(0)	3(3.4)	0(0)	0(0)	1(1.)
Mean±SD**	55.46±6.98	3.41±0.61	102.33±20.02	1.028±0.24	1.997±0.41	45.318± 6.02
CI 95***	53.99, 56.93	3.28, 3.54	98.12, 106.54	0.98, 1.079	1.91, 2.083	44.053, 46.583
P-value*		**0.292**			**0.734**	
**Chocolate Agar**						
**n**	86	87	87	86	87	87
**0**	80(93)	82(94.3)	76(87.4)	83(96.5)	85(97.7)	82(94.3)
**1-10**	6 (7)	5(5.7)	11(12.6)	3(3.5)	2(2.3)	5(5.7)
**11-100**	0(0)	0(0)	0(0)	0(0)	0(0)	0(0)
**>100**	0(0)	0(0)	0(0)	0(0)	0(0)	0(0)
**Mean±SD**	0.52±0.13	0.84±0.16	1.47±0.39	0.788±0.12	0.622±0.09	1.287±0.25
**CI 95**	0.492, 0.547	0.806, 0.874	1.388, 1.551	0.763, 0.813	0.603, 0.641	1.234, 1.340
P-value*	**0.215**				**0.488**	

* P<0.05- Kruskal -Wallis test

** SD= Standard deviation

*** CI: Confidence interval

In both povidone-iodine 5% and chloramphenicol groups, the mean colony formation in blood agar after the intervention decreased and based on the P-value obtained from the Wilcoxon test, these changes appeared to be significant (p = 0.032 and p = 0.005). However, in the Povidone-iodine 1% group, a reduction occurred in the mean of colony-forming units in the blood agar after the intervention, which was not significant (p = 0.548). In the chocolate agar, a fall in the number of colony-forming units was observed in all three groups, but it was statistically non-significant. (Table 3)

**Table 3:** Comparison of changes in the mean of colony forming units/mL (CFU/mL) before and after the intervention in the study groups

		Colony forming units/mL				
Study groups		Before intervention		After intervention		P-value*
		Mean	standard deviation	Mean	standard deviation	
**Povidone Iodine 5%**	Blood Agar	6.98	55.46	0.24	1.028	**0.032**
	Chocolate Agar	0.13	0.52	0.12	0.788	**0.510**
**Povidone Iodine 1 %**	Blood Agar	0.61	3.41	0.41	1.997	**0.548**
	Chocolate Agar	0.16	0.84	0.09	0.622	**0.553**
**Chloramphenicol**	Blood Agar	20.02	102.33	6.02	45.318	**0.005**
	Chocolate Agar	0.39	1.47	0.25	1.287	**0.150**

* P<0.05-Wilcoxon test

## Discussion

Endophthalmitis is a rare complication following eye surgery. Studies that tend to measure the effect of different prophylactic methods on postoperative endophthalmitis require scores of patients, and the process appears to be challenging.^10^, ^11^ We, therefore, considered the number of conjunctival bacteria as an indicator to assess postoperative endophthalmitis risk, as had been used in other studies.

The study's limitation was that we could not show a relationship between the reduction of positive cultures and the incidence of endophthalmitis. Because the incidence of endophthalmitis is low, at least 4000 patients are needed for the study. On the other hand, it was impossible to follow patients regarding postoperative endophthalmitis due to time constraints. Moreover, this research was carried out in a single-centre, which might have engendered bias. For this reason, we suggest a prospective longitudinal probe for further studies.

Evidence suggests that normal eyes' positive conjunctival bacterial culture rate varies between 60.9% and 100%.^12^ In a study by Li et al., the average rate of positive conjunctival culture was 33.33%.^13^ Also, in another study conducted by Lin et al., 256 eyes were examined, out of which 68 (26.6%) were positive.^8^ Moreover, in Tao's et al. study, positive conjunctival culture was reported in 24.2% of cases. In contrast, in the present study, the positive culture rate showed to be 18.85%, which is lower than the reported value in some previous studies.^14^

This discrepancy in the percentage of positive cultures in different studies may be attributed to various reasons, such as the degree of skill required in conjunctival sampling, the conditions for transferring samples to the laboratory, and the skill in culturing and isolating bacteria. However, the exact cause is yet to be known.^12^ In Azari's study 15, a positive culture rate was reported at 3%, and the researchers concluded that the reason for the high percentage of positive conjunctival culture in other studies is the contact of the applicator with the edge of the eyelid during sampling. In the present study, the most common microorganism identified was *Staphylococcus epidermidis*. Tao et al. (2017) also identified *coagulase-negative staphylococci* (CONs) as the most abundant microorganism (39.7%) in the conjunctiva.^14^ In the work by Bing li et al., CONs were the most common microorganisms, accounting for 77.4% of all bacteria grown in the thioglycollate medium.^13^ Moreover, as reported by the results of the research by Inagaki et al. as well as Ikuno et al., the most common microorganisms distinguished in the culture medium were *Staphylococcus epidermidis*, the species *Corynebacterium* and *Propionibacterium acne*.^2^, ^7^ Coskun et al. also reported the most common microorganisms in the conjunctiva as gram-positive cocci, but often CONs and *Staphylococcus aureus*.^12^ The results of the bacterial flora in the present study were similar to those of earlier researchers with other researchers' reports (Figure 2). In cases where the immune system is weak, including diabetes mellitus, ageing, or taking corticosteroids, the microbial flora and subsequent infection rates are expected to grow.^10^ In a study conducted by Ratnumnoi et al.^16^ in 2017, a significant difference was found in the number of conjunctival bacteria in diabetic patients compared to patients without diabetes (P=0.028). In another study by Moreno et al., the rate of positive culture in the diabetic group (83.3%) stood higher than in the non-diabetic group (80%). However, a statistically nonsignificant difference was identified between the two groups.^17^ Furthermore, in a study by Adam et al., no significant difference was observed in the bacteria isolated from the conjunctiva in diabetic and non-diabetic participants. Moreover, a non-significant difference was observed in the frequency of positive cultures between diabetic and non-diabetic subjects. However, the number of gram-negative bacteria was higher in the diabetic participants.^10^ According to the present study, no significant difference was observed in the number of positive conjunctival cultures between patients with a history of diabetes and non-diabetics (Table 1); this may be because all the patients had controlled diabetes and took medication.

Diabetes has also been shown to have little effect on the bacterial flora of the conjunctiva.^17^ Ratnumnoi et al. suggested ageing and diabetes as two factors associated with the growth of more bacteria in the conjunctiva.^16^ Tao's study showed the number of isolated bacteria to be higher in men than in women, especially in those over 65.^14^ Morento et al. also reported the bacteria level acquired from the conjunctiva of young adults (under 40 years) being lower than that of older persons.^17^ The results of this study, however, revealed that gender bears no role in the conjunctival flora culture (p-value = 0.677). Ratnumnoi et al. also reported that the amount of conjunctival flora enhances with age; that is, people 70 years of age and older prove to have more microbial flora^2^,^16^; no such association, however, was observed in our study (p-value = 0.447).

Using povidone-iodine as a preoperative prophylaxis for years has shown it to be a cost-effective solution with few side effects and the capacity to greatly reduce conjunctival bacteria.^18^ Inagaki et al. reported that using povidone-iodine 5% reduced the rate of positive conjunctival cultures from 50% to 16.7%.^2^ Moreover, predicated on Bing Li et al.'s results, Povidone-iodine effectively reduces the conjunctiva's bacterial flora, and patients well tolerate all its concentrations (1, 5 and 10%).^4^ In another study by Fan et al., using 0.05% povidone-iodine for 30 seconds, one and two minutes, all culture results appeared negative, and no endophthalmitis was reported after three months.^19^ Garg et al. also reported that using 5% preoperative povidone-iodine to wash the conjunctiva reduces the conjunctival flora by 57-60.8%.^20^

The present study was consistent with the previous studies in that after using 5% povidone-iodine, the positive culture rate decreased from 6.98% to 0.24%, and the changes were significant (p-value = 0.032). However, only a reduction of 6.74% was observed (Table 3). In the povidone-iodine 1% group, although the amount of conjunctival flora was reduced, it was not statistically significant. This is similar to the results of Gnanasekaran et al., where a significant decrease was found in CFU with a povidone-iodine concentration of 5% (p < 0.001). However, povidone-iodine concentrations lower than 5% were ineffective in reducing bacteria growth.^21^ In accounting for the ineffectiveness of povidone-iodine 1% in this study, it can be said that because the intervention was performed in the ward, only one drop of povidone-iodine was applied in the eye. No anaesthesia was also used.

These results indicate that the use of a low amount of povidone-iodine 1% fails to bear much effect on reducing the conjunctival flora; however, povidone-iodine 5% irritates the eye and, although not easily tolerated, even in small amounts but prolonged contact can effectively reduce the bacterial flora. Nevertheless, Kasper et al. reported that washing conjunctiva with a povidone-iodine solution is more effective than using only one or two drops of povidone-iodine.^22^ Antibiotics are used as prophylaxis before cataract surgery to reduce pathogens and microorganisms in the conjunctiva and eyelids; therefore, they should be broad-spectrum, have low toxicity on the cornea, and carry high bactericidal power.^12^ In the present study, topical chloramphenicol drops were used, the results of which demonstrated that after using the drop, the average number of CFU in blood agar decreased from 20.02 to 6.02 (Table 3), i.e., a 14-unit reduction; this was however higher than the two previous groups.

Coskun et al. conducted a study uncovering that ciprofloxacin 0.3% is more effective in reducing conjunctival bacterial flora than ofloxacin 0.3% and povidone-iodine 5%; however, no significant difference was observed between povidone-iodine 5% and ofloxacin groups in terms of reduction in positive culture after an intervention.^12^ Bing Li et al. also used levofloxacin 0.3% four times daily before surgery, which showed that it effectively reduces conjunctival bacteria. However, the effect appears to be more significant by washing conjunctival with povidone-iodine.^4^ In the study of Sorkhabi et al., no difference was reported in the mean reduction of colony-forming units after using povidone-iodine 5%, ciprofloxacin, and normal saline,^9^ thus not being consistent with the results of the present study.

## Conclusion

In this study, applying topical chloramphenicol or 5% povidone-iodine was an effective and acceptable method for reducing bacterial flora, although chloramphenicol was effective.
